# Calcifying Acne: An Unusual Extraoral Radiographic Finding

**DOI:** 10.1155/2017/3514936

**Published:** 2017-11-02

**Authors:** Thomas Horgan, Catherine McNamara, Anthony Ireland, Jonathan Sandy, James Puryer

**Affiliations:** ^1^Bristol Dental School, Lower Maudlin Street, Bristol, BS1 2LY, UK; ^2^HSE Regional Orthodontic Department, St. James's Hospital, Dublin 8, Ireland

## Abstract

Calcinosis cutis is a condition of accumulation of calcium salts within the dermis leading to the formation of a calcified mass. This complication has been reported in acne vulgaris and other systemic metabolic disorders. This paper presents a rare case of calcinosis cutis in a 14-year-old male which was found at a routine orthodontic assessment.

## 1. Introduction

Acne vulgaris (AV) is a benign dermatological condition resulting from overproduction of sebum by the skin's sebaceous glands. The condition commonly occurs in adolescents and young adults. It primarily affects the face, upper back, and chest, producing two types of lesions, inflammatory lesions and comedones. Physical sequelae of the condition such as scar formation and hyperpigmentation are common, but rarer sequelae such as cutaneous calcifications are infrequently reported in the literature [1, 2].

Calcinosis cutis occurs as a result of the deposition of calcium salts in the dermis and subcutaneous tissues. This complication has not only been reported in AV and systemic metabolic disorders such as hyperparathyroidism, infection, or connective tissue disorders but can also occur following trauma to an area of the skin. Calcinosis cutis can be subdivided into 4 groups based on aetiology: dystrophic, metastatic, iatrogenic, and idiopathic [3]. The dystrophic type occurs in patients with normal calcium and phosphate levels but confined to areas where tissue has been damaged following infection, inflammation, or connective tissue diseases.

This paper presents a case report of dystrophic calcinosis cutis in a 14-year-old male, resulting from inflammatory facial acne.

## 2. Case Report

**Figure 1:**
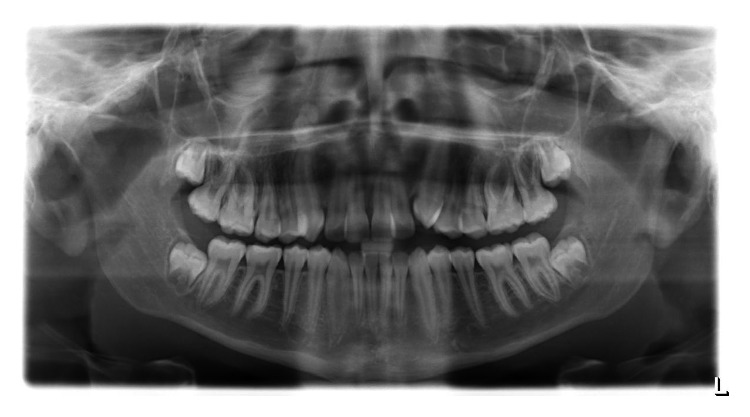
OPG radiograph taken prior to orthodontic treatment showing a calcified mass above the apex of the upper right canine.

A Caucasian male aged 14 years attending an orthodontic clinic for a routine assessment presented with an incidental radiographic finding. A well-defined calcified mass was visible at the apex of the maxillary right permanent canine on the orthopantomogram (OPG) radiograph (Figure 1).

**Figure 2:**
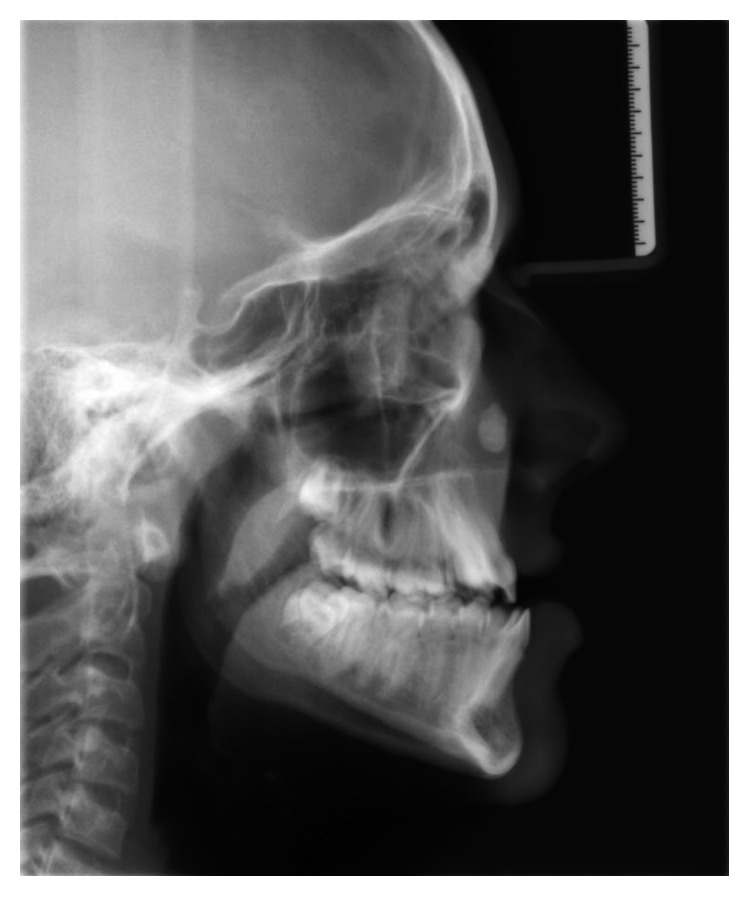
Lateral cephalometric radiograph taken prior to orthodontic treatment showing a calcified mass above the apex of the upper right canine.

A lateral cephalometric radiograph showed that this mass was significantly superior to the dentition in the antral inferior nasal region (Figure 2). The mass was a well-defined spherical lesion, measuring 1.5 by 1 cm with a radiopaque, homogeneous internal structure. It lay superior to the canine fossa and anterior to the maxillary sinus anterior wall.

**Figure 3:**
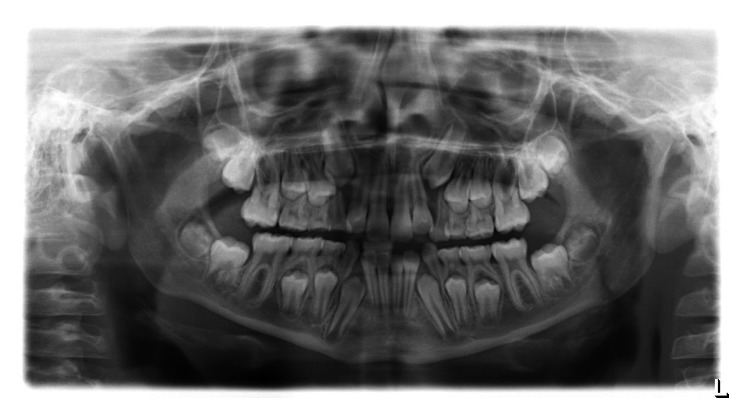
OPG radiograph taken 4 years previously with no evidence of the calcified mass seen on the later radiographs.

This calcified mass had not been present on the OPG radiograph taken previously, at age 10 years (Figure 3).

From the age of 12, it was reported that the patient suffered with facial AV. He attended a consultant dermatologist who treated the condition successfully. The calcified mass was previously assessed at dermatological review. Despite the fact that it was a solitary lesion, it was diagnosed as dystrophic calcinosis cutis. The dermatologist advised no intervention as there was a significant risk of facial scarring if surgically removed. The mass was asymptomatic and presented little risk of infection. The patient was placed on annual review with his dermatologist.

Although the mass was palpable and mobile, the patient's dermis was intact and acne-free. The mass was not visible clinically on extraoral examination. Facial profile and contour were normal. Orthodontically, he presented with a Class III incisor relationship on a Class III skeletal base with crowding in the maxillary arch. Both the patient and his parents were given the option of orthognathic surgery to treat his malocclusion and underlying significant skeletal relationship discrepancy. After extensive discussions and reviews with a maxillofacial surgeon, they opted for orthodontic treatment only, namely, alignment of the maxillary arch teeth on a non-extraction basis, followed by permanent retention. The orthodontic treatment was uneventful and successful.

## 3. Discussion

Acne vulgaris is a dermatological disease caused by changes in the hair follicle and its associated sebaceous gland, jointly called the pilosebaceous unit. AV affects approximately 80% of the population between 12 and 25 years of age. It does not display race or gender prevalence differences [4]. Three factors are required for the development of AV, these being sebum, androgens, and the bacterium *Propionibacterium acnes*. AV begins with the release of androgens, which in turn leads to the increased production of sebum in the sebaceous glands and intrafollicular hyperkeratosis [4]. The resulting skin lesions can be either comedones or inflammatory in nature.

Comedones are subdivided into blackheads and whiteheads. A blackhead is a comedone which is open to the skin surface allowing the contents to escape. The black colour is due to melanin pigmentation. The whitehead is a closed comedone, which does not allow its contents to escape.

Inflammatory lesions arise if the walls of a closed comedone rupture. Lipoid tissue is expressed into the surrounding dermis. This sets up a foreign body inflammatory reaction and coupled with *P. acnes* leads to infection and the formation of acne lesions including papules, pustules, nodules, and/or cysts [5].

Healing of the AV inflammatory lesions can occur via two processes. Firstly, healing by fibrous tissue can lead to scar formation, while secondly, the epidermis portion of the remaining comedone walls sends out sheaths of epithelium to encapsulate any inflammatory material. The encapsulated mass can become thickened by the evaporation or absorption of fluid. This thickened mass, coupled with necrotic tissues, which are produced as part of healing, provides an ideal environment for the formation of calcifications known as calcinosis cutis [4–6].

Calcinosis cutis is characterized by abnormal deposits of calcium salts in the dermis and/or hypodermis. It often presents as multiple hard pale plaques, nodules, or papules; however, it can present as a singular lesion also [4, 5]. Based on its aetiology, it is divided into 4 subtypes: dystrophic, metastatic, iatrogenic, and idiopathic.

Dystrophic calcinosis cutis occurs in areas of tissue damage secondary to infection, inflammatory processes, connective tissue diseases, or cutaneous neoplasms [7]. It is the most common form of ectopic calcification and develops around local tissue damage without any alteration to calcium or phosphate metabolism, for example, in AV. In contrast, metastatic calcification is due to changes in the metabolism of calcium or phosphate, which leads to precipitation of calcium in the skin [3]. Idiopathic calcification arises without any underlying tissue damage or metabolic disorder, while iatrogenic calcification is secondary to medical interventions which can damage tissue or lead to disturbances in calcium and phosphate metabolism. An international multicenter cohort study [8] found an overall frequency of calcinosis cutis of 22% in patients with connective tissue diseases; however, there are no specific data relating to incidence of dystrophic calcinosis cutis in the literature [8].

Dystrophic cutaneous calcification secondary to long-term acne was first reported in 1928 by Hopkins [9] and later by Leider [10] who found radiographic evidence of calcinosis cutis in four of six patients with long-term AV. Other medical studies have shown that these calcifications can be identified in as many as half of all cases of long-term AV (i.e., 7 years or more) [11, 12]. Interestingly, the patient in our case report had only suffered with AV for 2 years. In addition, it is the only reported case of dystrophic calcinosis cutis reported in the orthodontic setting.

Diagnosis of dystrophic calcinosis cutis is based on serological investigations, suitable imaging, and biopsy if required [1, 2, 4]. Histopathological examination would show a focus of a well-circumscribed, round, basophilic substance, which would stain black with the Von Kossa stain. It may be located in the upper dermis, surrounded by thick collagen fibers and sometimes by epithelioid and multinucleated giant cells [13].

Due to its radiopaque nature, dystrophic calcinosis cutis can be visualized on plain film radiographs; however, other useful modalities which could have been used include cone beam computed tomography (CBCT) and ultrasound [1]. These imaging modalities can be used either singularly or in combination to improve localization; however, if CBCT is to be used, one must consider the effects of a higher radiation dose [1, 2]. In addition to serological assessment for connective tissue disease, full biochemical assessment is required to exclude any abnormality of systemic calcium homeostasis—this should include serum calcium, phosphate, alkaline phosphatase, vitamin D, and parathyroid hormone (PTH) estimation [14]. Differential diagnoses include osteoma cutis, calcified lymph nodes or cysts, and areas of calcified necrotic materials such as caseous granulomas in tuberculosis. While dystrophic calcinosis cutis results in calcification of areas in the skin, osteoma cutis causes ossification of the dermis and subcutaneous tissue. It can be deemed that primary ossification occurs in the absence of a preexisting cutaneous disorder, while secondary osteoma cutis occurs when bone forms in a preexisting lesion [15].

In cases of diffuse calcinosis cutis, such as metastatic or iatrogenic, medical management is required to correct the systemic imbalance. This includes the use of warfarin, bisphosphonates, minocycline, ceftriaxone, diltiazem, aluminium hydroxide, probenecid, intralesional steroids, and intravenous immunoglobulin [14]. For cases of singular lesions, such as that highlighted in our case, surgical treatment is preferred. Methods include surgical excision, curettage, laser therapy, and lithotripsy [16]. Surgical management in this case report was contraindicated, given the risk of facial scarring postoperatively. The mass was asymptomatic and considered at low risk of reinfection. Facial profile and dermal contour were normal. The location of this calcified mass would have posed a problem, however, had the patient opted for orthognathic surgery for his Class III malocclusion. As both he and his parents opted for a conservative orthodontic approach, no surgical intervention was necessary, and annual observation of this lesion by his dermatologist continued.

## 4. Conclusions

Dystrophic calcinosis cutis can occur as a sequela of long-term AV or alongside underlying metabolic disorders. Dentists should be aware of this phenomenon, as it can occur in the facial region and may present as an incidental finding on routine dental radiographs, both intra- and extraoral.
